# Next generation sequence analysis of the transcriptional response to neonatal hyperoxia

**DOI:** 10.1186/1755-8166-7-S1-P85

**Published:** 2014-01-21

**Authors:** Soumyaroop Bhattacharya, Zhongyang Zhou, Min Yee, Ashley Lopez, Valarie Lunger, Bradley Buczynski, Gloria Pryhuber, Thomas Mariani, Michael OReilly

**Affiliations:** 1 Division of Neonatology, Department of Pediatrics, University of Rochester Medical Center, Rochester NY, USA

## Background

Bronchopulmonary Dysplasia (BPD) is a major complication of preterm birth associated with significant morbidity. BPD is a debilitating condition characterized by inflammation, enlarged airspaces, vascular dysmorphia and aberrant extracellular matrix accumulation that is typically described as arrested lung development. Rodent models involving neonatal exposure to excessive oxygen concentrations (hyperoxia) have been used to study the mechanisms contributing to BPD pathology. Transcriptomic assessment of the effects of hyperoxia in neonatal mouse lungs using RNASeq will help to identify genes and pathways associated with BPD.

## Materials and methods

Whole lung tissue from newborn C57BL/6 mice exposed to 100% oxygen for 10 days (n=8) and room air-exposed age matched controls (n=6) were compared. Total RNA was isolated from individual whole lung tissues (n=14) and pooled in duplicates to perform transcriptome Sequencing (RNA-seq). Alignments were generated using multiple algorithms (CASAVA; TopHat; and SHRiMP). Raw counts obtained from each alignment algorithm (using HT-Seq) were further and filtered to remove undetected genes. Differentially expressed genes were detected using Significance Analysis of Microarrays (SAM) and CuffDiff2, on each version of mapped and normalized data. Ingenuity Pathway Analysis (IPA) was used for pathway and network analyses. Expression patterns for selected genes were examined by quantitative polymerase chain reaction (qPCR).

## Results

248 genes were identified as differentially expressed between hyperoxia and control samples by both SAM (median FDR = 0) and CuffDiff2 (p<0.05) and had a fold-change ≤ 2. We successfully validated 17 of 24 genes by qPCR. Canonical pathways significantly dysregulated in hyperoxia lungs included Nrf2-mediated oxidative stress signaling, p53 signaling, hepatic fibrosis and sildenafil pathways. Interestingly most genes significantly affected following hyperoxia exposure (~70%) showed a pattern of expression consistent with an arrest in lung development. A subset of the genes dysregulated in hyperoxic neonatal mouse lungs, were also differentially expressed in human BPD lung tissue.

## Conclusions

We have identified genes dysregulated in mouse of BPD-like pathology. Further analysis of these data will enhance our current knowledge of BPD, and may be useful for developing novel therapeutic strategies.

